# Use of Gracile and semi-tendinosus tendons (GRAST) for the reconstruction of irreparable rotator cuff tears

**DOI:** 10.1186/s12891-021-04197-6

**Published:** 2021-04-05

**Authors:** Marie Protais, Maxime Laurent-Perrot, Mickaël Artuso, M. Christian Moody, Alain Sautet, Marc Soubeyrand

**Affiliations:** 1 Département de chirurgie orthopédique et traumatologique – Hôpital Saint-Antoine, Assistance Publique – Hôpitaux de Paris (APHP), 184 rue du faubourg Saint Antoine, 75012 Paris, France; 2 Department of Hand, upper extremity and microsurgery, Prisma Health System, Greenville, SC USA; 3 Unité de chirurgie du membre supérieur, Clinique Saint Jean l’Ermitage, 272 avenue Marc Jacquet, 77000 Melun, France

**Keywords:** Irreparable rotator cuff tear, Arthroscopy, Gracilis, Semitendinosus

## Abstract

**Background:**

Irreparable rotator cuff tears are common and difficult to treat. Techniques for “filling the loss of substance” require fixation to the rotator cuff stump (tendon augmentation) or to the glenoid (superior capsular reconstruction), which are complicated by the narrow working zone of the subacromial space. The main objective of this study was to determine whether a braided graft of gracilis (GR) and semitendinosus (ST) could fill a loss of tendon substance from an irreparable rupture of the supra- and infraspinatus, by fixing the graft to the greater tuberosity and the spine of the scapula.

**Methods:**

This was a cadaveric study with the use of ten specimens. The GRA and ST tendons were harvested, braided and reinforced with suture. An experimental tear of the supraspinatus (SS) and upper infraspinatus (IS) retracted at the glenoid was made. The GRAST transplant was positioned over the tear. The transplant was attached to the greater tuberosity by two anchors and then attached to the medial third of the scapular spine by trans-osseous stitching. The percentage of filling obtained was then measured and passive mobility of the shoulder was assessed. We proceeded to the same technique under arthroscopy for a 73 years old patient whom we treated for a painful shoulder with irreparable cuff tear. We inserted a GRAST graft using arthroscopy.

**Results:**

The Braided-GRAST allowed a 100% filling of the loss of tendon substance. Mobility was complete in all cases.

**Conclusion:**

This technique simplifies the medial fixation and restores the musculo-tendinous chain where current grafting techniques only fill a tendinous defect. The transplant could have a subacromial “spacer” effect and lower the humeral head. The donor site morbidity and the fate of the transplant in-vivo are two limits to be discussed. This anatomical study paves the way for clinical experimentation.

## Background

The rotator cuff has two functions: to rotate the head of the humerus (and consequently the whole upper limb) in the three planes of space [6] and to coapt the humeral head against the glenoid [8] .

With aging, degenerative tears are frequently encountered. This cuff tear generally begins in the anterior part of the supraspinatus and then gradually extends to the infraspinatus [2].

**Fig. 1:**
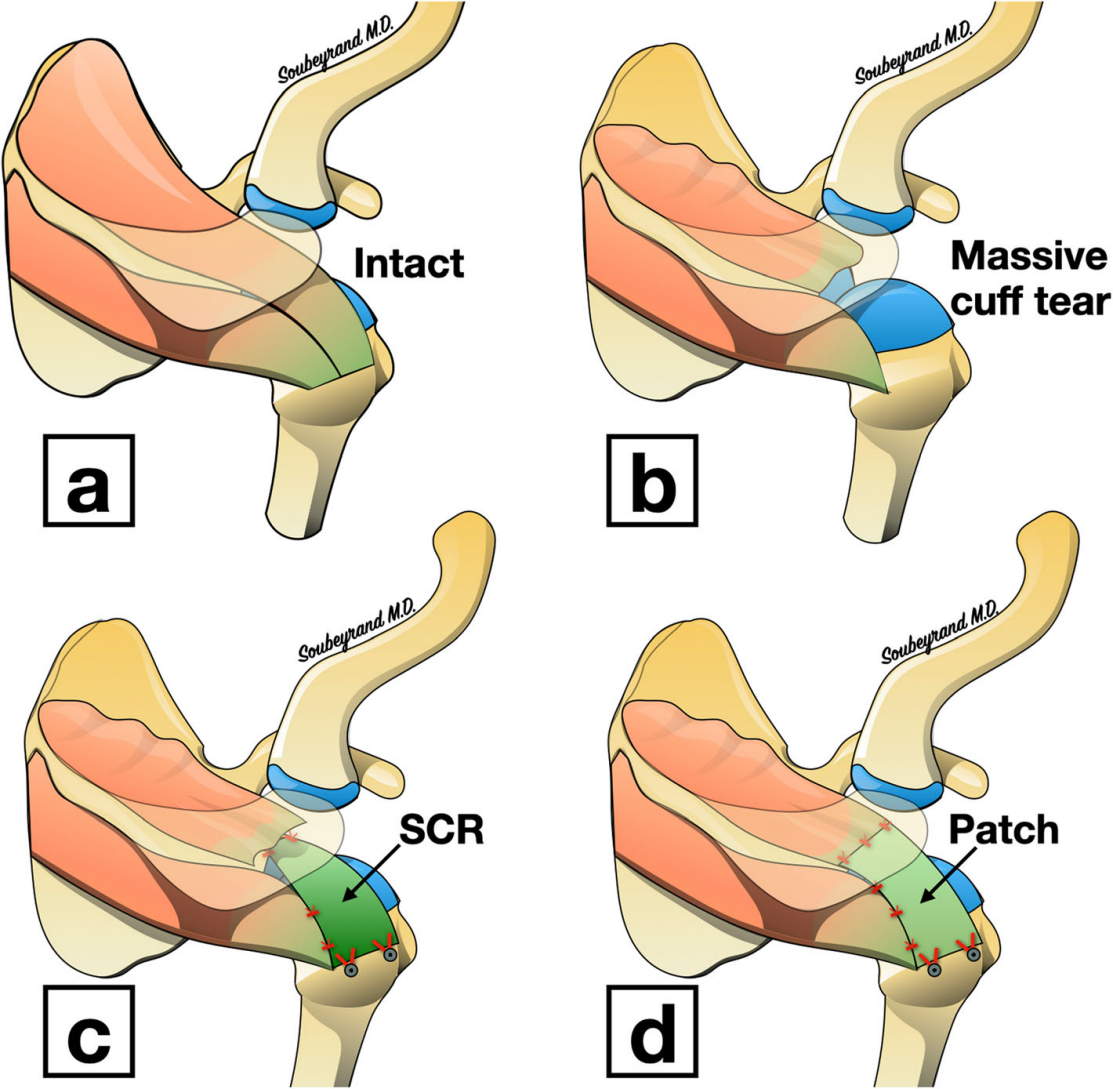
Intact rotator cuff (**a**), rotator cufftear (**b**) and the different options for filling the tear: Superior Capsular Reconstruction (SCR) (**c**) and the interposition patch (**d**) (images belong to the author Marc Soubeyrand)

Due to the effect of muscular contraction, the tendon stump progressively retracts towards the glenoid. If not used, the muscle then undergoes atrophy and fatty degeneration [13]. When fatty infiltration and retraction are too great, it is no longer possible to bring the ruptured tendon back to its humeral insertion: the tendon is then irreparable [11]. Different surgical options then exist, some of which consist of filling the loss of tendinous substance. These filling techniques are technically complex, especially when performed using arthroscopy. In fact, they require fixation to the cuff stump (tendon augmentation) [4, 20, 21] or to the glenoid (superior capsular reconstruction) [14] in a subacromial space that is often times quite narrow (Fig. 1).

Some authors have proposed to perform this filling either with autologous grafts such as fascia lata [12], quadricipital tendon [20], allografts [10], xenografts [15] or synthetic matrices [3].

However, these different grafts have important limitations such as the morbidity of the harvest, the inadequate thickness of the graft, the immunological or infectious risk, and the cost.

In order to overcome these limits, we propose a reconstruction technique of the cuff which is original for two reasons.

The first lies in the choice of grafts used and their preparation method. We have chosen to combine the use gracilis (GRA) and semitendinosus (ST) tendons by braiding them to form the GRAST graft. The removal of these tendons has low morbidity and they are usually sizable [1]. The aim of braiding is to construct a graft thickness comparable to the cuff.

The second point of originality is the attachments locations of the graft from the humerus to the spine of the scapula. This avoids complex fixation procedures to the stump or glenoid in the narrow subacromial space. Fixation to the scapular spine is also technically less demanding because it is practically subcutaneous.

The main objective of this study was to determine whether a GRAST transplant can fill a tendon defect from an irreparable rotator tear of the supra- and infraspinatus, while being long enough to reach the spine of the scapula and the greater tuberosity of the humerus.

## Methods

This was a cadaveric study on ten specimens. The mean age at death was 78 years (72–91). Exclusion criteria were the presence of shoulder stiffness, deformity or signs of prior surgery. The study protocol was approved by our institutional review board (Comité Scientifique et Ethique de l’APHP) and was registered under the CNIL (Commission Nationale de l’Informatique et des Libertés) number 13810*01. Anatomical subjects were used in accordance with the willingness to donate one’s body for teaching and medical research, expressed in writing during the donor’s lifetime. The subjects came from the Fer à Moulin Institute, 7 rue du Fer à Moulin, 75005, Paris, France, Assistance Publique des Hôpitaux de Paris (APHP). We proceeded to the same technique under arthroscopy for a 73 years old patient whom we treated for a painful shoulder with irreparable cuff tear. We inserted a GRAST graft using arthroscopy. The participant gave her informed written consent for the surgery, the submission and the publication of images in Fig. 6a, b, c, d, e.

### Experimental protocol

**Fig. 2:**
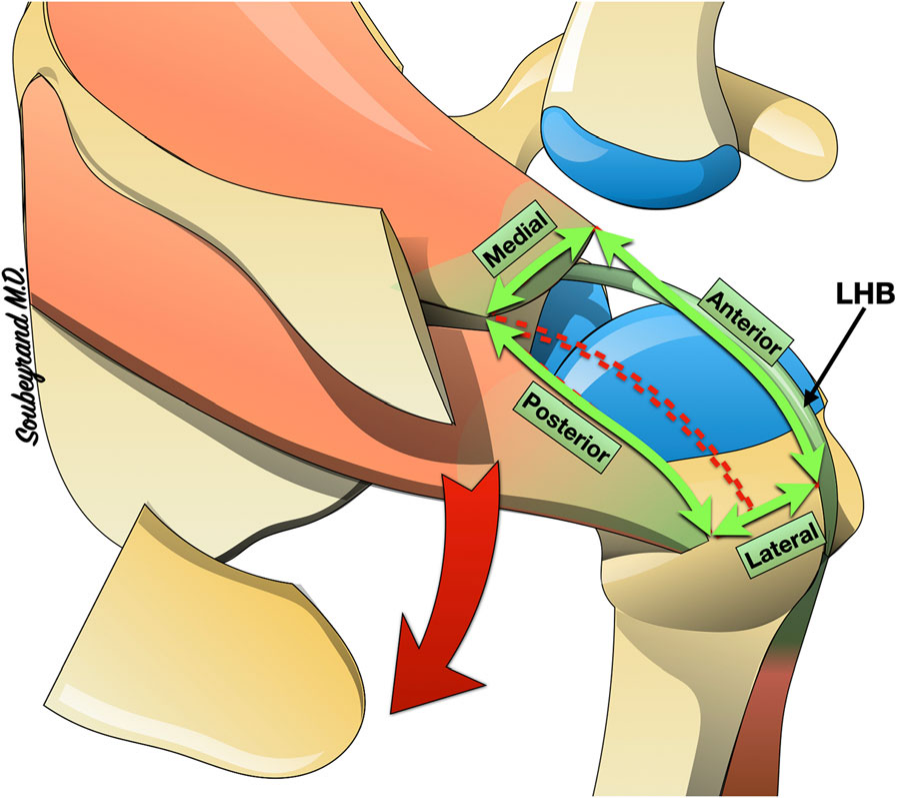
Experimental protocol: the acromion is removed. An irreparable tear of the cuff is simulated by excising the entire supraspinatus tendon and the upper half of the intraspinatus tendon. LHB: Long Head of the Biceps (images belong to the author Marc Soubeyrand)

Subject was seated in a beach chair position, one shoulder and one knee in the field. The GRA and the ST were first harvested (Fig. 2) through a vertical skin incision, 2 cm inwards and 2 cm below the tibial tuberosity. The GRA and ST were identified below the sartorius fascia and then harvested using a tendon stripper.

Once the two tendons had been harvested, they were placed parallel to each other and folded in half so that both tendons were the same length on each side. The result was four strands and a plication. Two traction sutures (Fibertape® and TigerTape®, Arthrex) were placed one centimeter apart and passed through the plicature. The graft was prepared by braiding the 4 strands to obtain the final GRAST graft.

The dry GRAST was then measured with a digital caliper: thickness, width, length and dry volume. The transplant was then preserved in a saline cup.

After 10 min of soaking in saline, the same dimensions were measured again on the wet GRAST.

The shoulder was then approached by a superolateral approach to perform an acromionectomy first. The experimental tear was then induced by excising the entire supraspinatus tendon and the anterior part of the infraspinatus from their humeral insertion to the glenoid, thus simulating an irreparable tear. The dimensions of this defect were measured: antero-posterior, medio-lateral (Fig. 2).

**Fig. 3:**
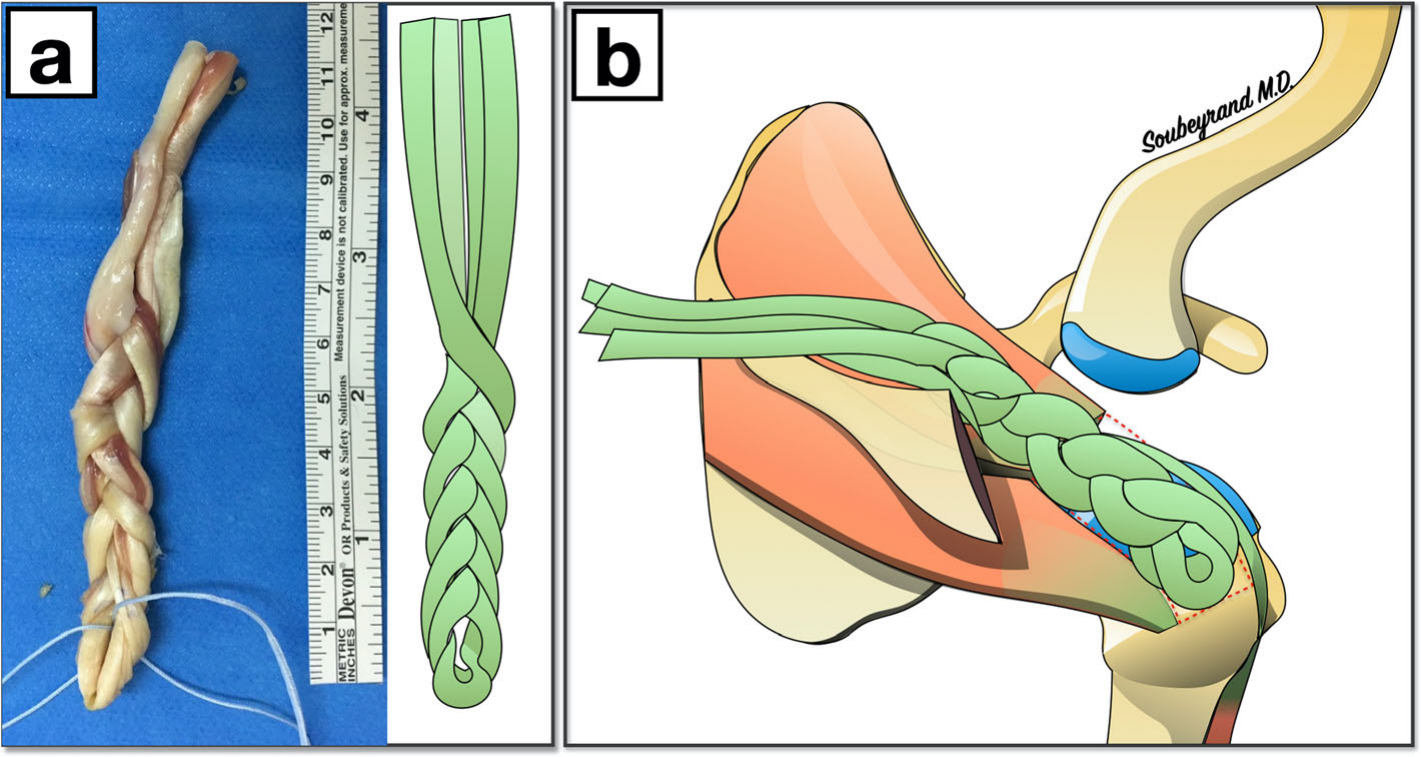
**a** The GRAST graft (**b**) Positioning of the GRAST graft over the tear zone of the cuff and in the supraspinatus fossa. (images belong to the author Marc Soubeyrand)

**Fig. 4:**
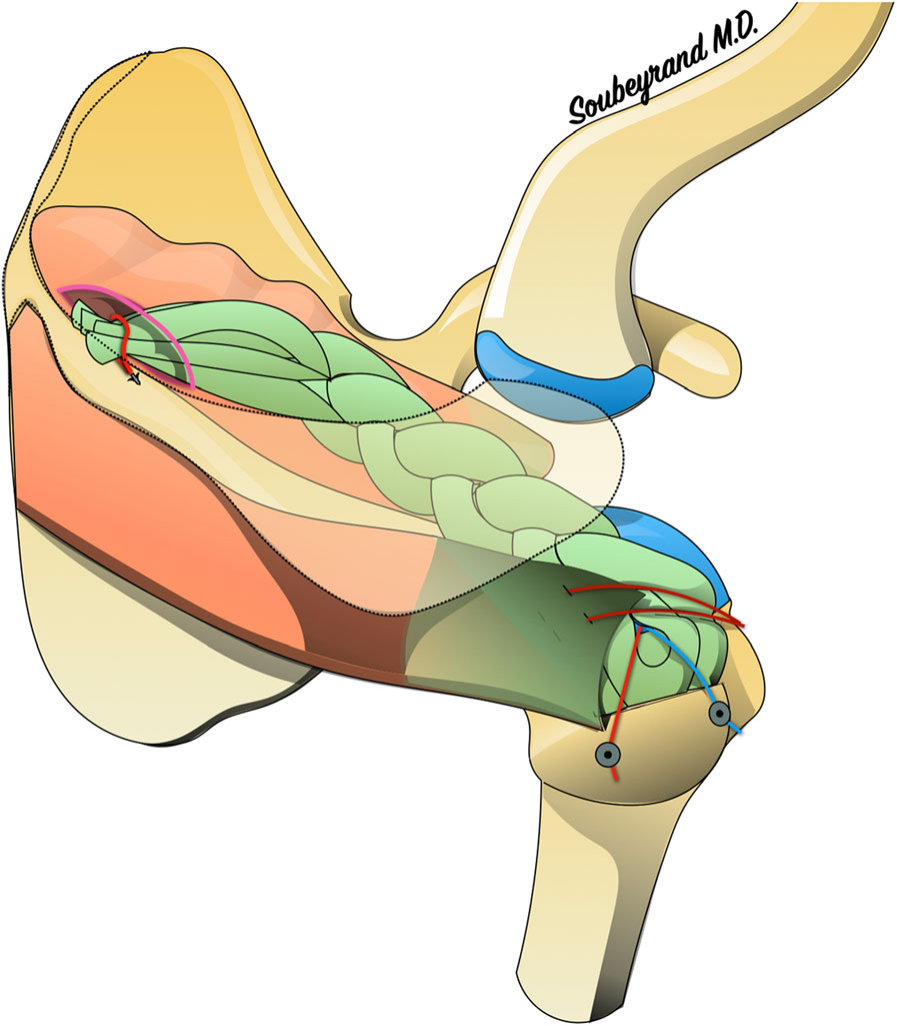
The principle of the GRAST technique. The GRAST is fixed to the humerus and the residual infraspinatus tendon is fixed over the GRAST. The other end of the GRAST is attached to the scapular spine. (images belong to the author Marc Soubeyrand)

The GRAST transplant was then positioned over the loss of substance and fixed with two anchors to the great tuberosity, as in a single-row repair. A suture is then passed from the remaining infraspinatus tendon and attached to the graft allowing the graft to cover the entire empty footprint left by the tear as well as incorporate the intact infraspinatus tendon into the repair. (Fig. 3). The medial end of the graft was brought to the point of fixation on the spine of the scapula. It was considered long enough if the graft was at least 1 cm longer than the point of fixation on the spine. This excess length of 1 cm seemed to us to be the minimum length necessary for correct scapular fixation (Fig. 4).

At the end of the procedure, the loss of residual material from the rotator cuff not covered by the graft was evaluated, as well as the percentage of antero-posterior and medio-lateral filling.

Each measurement was repeated twice by two double-blind operators.

## Results

The average length of the semitendinosus (ST) was 263 mm (σ = 28, min = 210, max = 300), that of the gracilis was 240 mm (σ = 34, min = 200, max = 260).

The dry graft averaged 114 mm long (σ = 18, min = 80, max = 130), 13.6 mm wide (σ = 1.26, min = 12, max = 16), and 5.8 mm thick (σ = 2,8, min = 4,7, max = 11).

The average hydrated transplant was 110 mm long (σ = 21, min = 86, max = 136), 15.6 mm wide (σ = 2.98, min = 11.7, max = 18.7), 7.7 mm thick (σ = 3.28, min = 4.7, max = 12.4). When its dimensions were reduced to a parallelepiped, there was an 85% increase in volume. (Table 1).

**Table 1:** Characteristics of the Braided GRAST

	Before soaking in saline	After soaking in saline
Lengh (mm)	114 (σ = 18, min = 80, max = 130)	110 (σ = 21, min = 86, max = 136)
Width (mm)	13,6 (σ = 1,26, min = 12, max = 16),	15,6 (σ = 2,98, min = 11,7, max = 18,7)
Thickness (mm)	5,8 (σ = 2,8, min = 4,7, max = 11)	7,7 (σ = 3,28, min = 4,7, max = 12,4)
Total volume (mm3)	8227 (σ = 1952, min = 5472, max = 11,440)	15,203 (σ = 10,800, min = 4069, max = 30,042)

The cuff tear was 25 mm wide (σ = 81, min = 18.4, max = 40) and 34 mm long (σ = 28, min = 30, max = 39.3), i.e. an area of 853 mm2 (σ = 238, min = 647, max = 1225) in a rectangle (Table 2). The braided graft filled the entire tear for all the cadavers, and the transplants was passing through the remaining cuff.

**Table 2:** Characteristics of the rotator cuff tear

Lengh (mm)	25 (σ = 81, min = 18,4, max = 40)
Width (mm)	34 (σ = 28, min = 30, max = 39,3)
Surface (mm)	853 (σ = 238, min = 647, max = 1225)

In all cases the graft length was sufficient to connect the major tuberosity of the humerus and the spine of the scapula.

During the arm elevation manoeuvres we observed that the GRAST was driven by the humerus towards the supraspinatus fossa. Its passive mechanical behavior therefore does not seem to impinge on the greater tuberosity under the acromion.

The procedure under arthroscopy was simple and took 74 min in total. We encountered no difficulty for the harvesting and the preparation of the graft. We inserted under arthroscopy the braded GRAST into the supraspinatus fossa throw a posteromedial approach via a short incision on the spine and then threaded medially to laterally through the supraspinatus stump using the strips that serve as tractor strands. These same strips were finally used for attachment to the major tuberosity by two anchors. The tension was simply adjusted when attaching to the spine under visual control, with the arm along the body. In addition, a tenotomy of the biceps and an acromioplasty were performed.

## Discussion

Our study shows that it is possible to completely fill the void left by an irreparable rotator cuff tear with a GRAST graft. The braiding of the GRAST makes it possible to reproduce the morphology of the torn tendon in all three dimensions of the space, all the better when the GRAST is hydrated (as is the case once it is implanted, especially under arthroscopy). The GRAST graft was able to connect the greater tuberosity of the humerus to the spine of the scapula in all cases. Finally, the passive mechanical behavior of the GRAST does not interfere with the elevation of the arm.

Our technique offers an intermediate solution between superior capsular reconstruction, which has become the primary treatment in many centers in the management of irreparable posterosuperior tears [14], along with partial repair with medialization [5] when the patient is not eligible for a reverse shoulder arthroplasty or tendon transfer. It simplifies medial fixation and restores the musculo-tendinous chain where current grafting techniques are limited with completely filling a tendon defect. In fact, extending the graft to the supraspinatus fossa also offers the hope of incorporation into the body of the muscle.

**Fig. 5:**
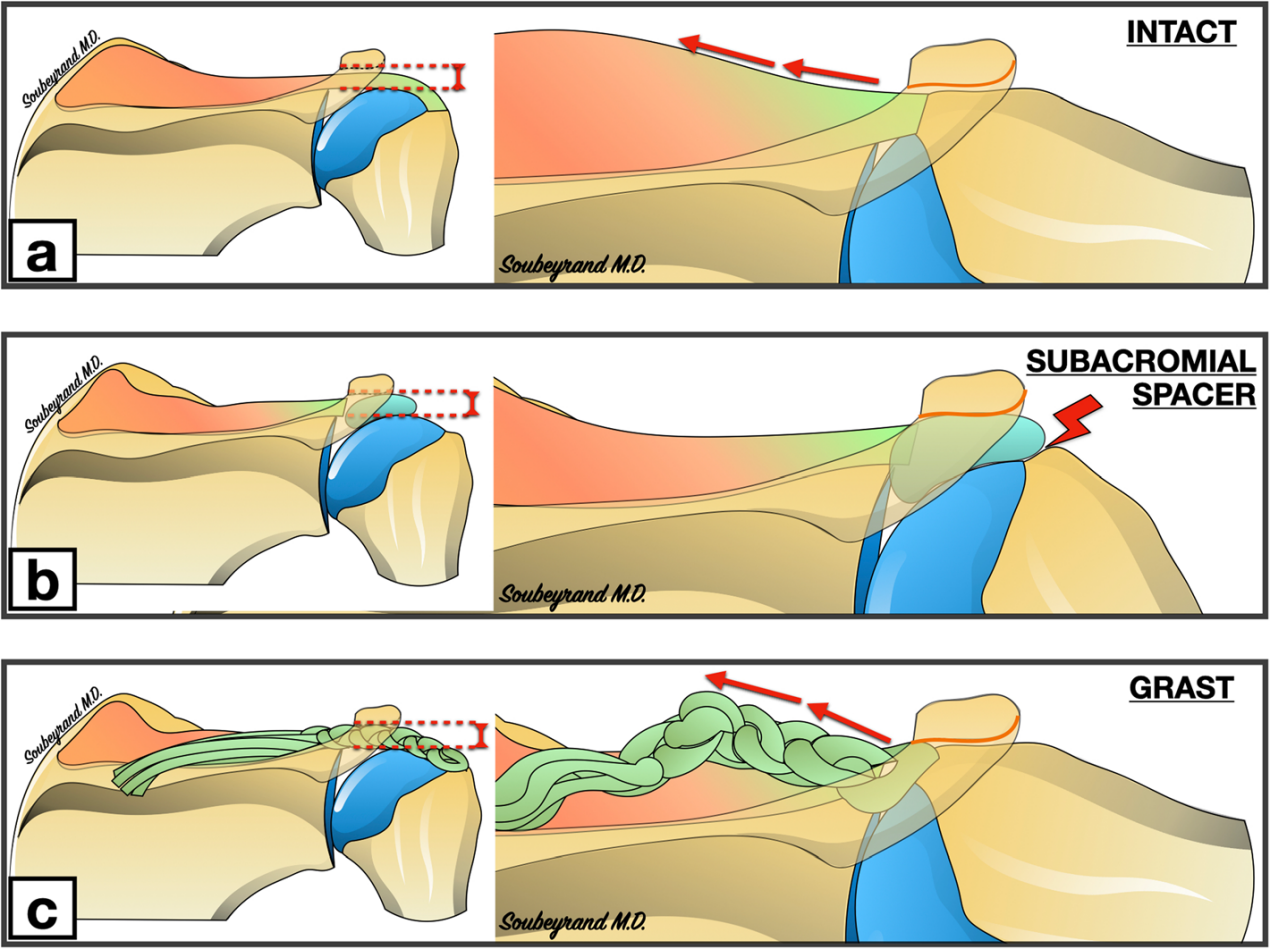
The subacromial height (red dotted lines) must be maintained when the arm is alongside the body. However, when the arm is raised, the greater tuberosity abuts the lower edge of the acromion and the subacromial height becomes almost zero. **a** When the arm is in neutral position next to the body, the supraspinatus tendon helps to maintain the subacromial height. When the arm is raised, the tendon escapes towards the supraspinatus fossa, leaving the place for the greater tuberosity. **b** Example of the subacromial spacers that maintain subacromial height when the arm is alongside the body. They may interfere with the course of the greater tuberosity when the arm is elevated because they remain in the subacromial space. **c** the GRAST graft has a mechanical action similar to that of the supraspinatus tendon. (images belong to the author Marc Soubeyrand)

Centering the head of the humerus in the glenoid is an essential prerequisite for the proper function of the shoulder, especially with forward elevation. Indeed, this allows the center of rotation of the humeral head to be maintained in an optimal position so that contraction of the deltoid causes rotation of the humeral head rather than proximal migration. In addition, it induces a tension effect on the deltoid fibres, which potentiates its strength when the elevation movement is initiated. The natural tendency of the humeral head is to migrate proximally under the action of the powerful deltoid. In the event of a massive tear in the cuff, this results in a reduction of the subacromial space. Since glenohumeral congruence is poor, only soft tissues (ligaments, tendons) can oppose this migration. As in many other anatomical sites, there are two complementary solutions to prevent migration of a bone piece in a given direction: “traction” in the opposite direction and “abutment”. Traction is usually based on a ligament and/or tendon whose force vector is in the opposite direction to the movement to be prevented. This is similar to the cable of an anchor that prevents the boat from drifting under the action of the current. The stop, on the other hand, consists of placing an obstacle that will oppose the migration of the bone piece. In the shoulder, the only stop between the humeral head and the acromion is the supraspinatus tendon. It has therefore been suggested that proximal migration of the humerus could be prevented by sliding a subacromial spacer implant. This is the case for example with subacromial balloons [16, 17]. However, when the arm is raised, the greater tuberosity will engage under the acromion and the subacromial space will become almost obsolete. If this space is already occupied by a subacromial spacer, the course of the greater tuberosity will be blocked because it will come into abutment with the spacer. The same structure that keeps the humerus at a distance from the acromion when the arm is in the lowered position must therefore be able to be gradually moved out of the way when the arm is raised, without blocking the course of the greater tuberosity. This is exactly what the supraspinatus tendon does. Our study confirms that during passive elevation of the arm, the GRAST graft gradually escapes from the subacromial space into the supraspinatus fossa. (Fig. 5).

However, this study is a cadaveric study and therefore has certain limitations.

**Fig. 6:**
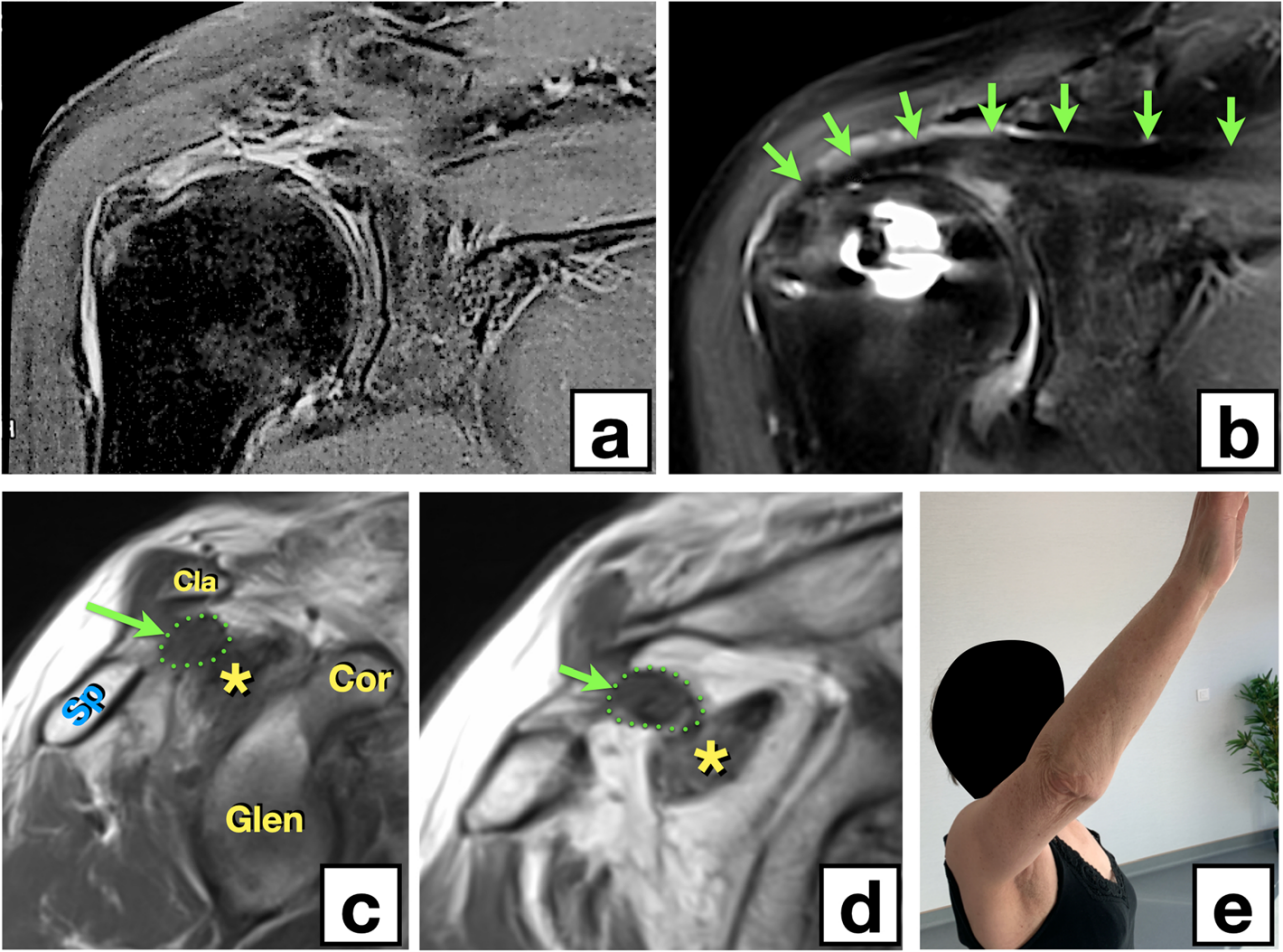
Clinical case. **a** Preoperative MRI showing the fully retracted tear of the supraspinatus. **b** MRI at 1 year postoperatively showing the incorporated GRAST graft (green arrows) from the humerus to the supraspinatus fossa. **c**, **d** Sagittal sections showing the GRAST graft in the supraspinatus fossa (green dotted line). Asterisk: amyotrophic supraspinatus. Sp: Spine of the scapula. Cla: Clavicle. Cor: Coracoid. Glen: Glenoid of the scapula. **e** Anterior elevation of the arm

As with any anatomical study, the question of in vivo transposition arises. Indeed, one legitimate question is the clinical tolerance of such a transplant as well as the biological fate of the transplant. It is obviously too early to report the results of a clinical series, but a first look at early results can be seen with the case of a 73 years old patient whom we treated for a painful shoulder with irreparable cuff tear. We inserted a GRAST graft using arthroscopy. At 1 year’s follow-up, the graft appeared well incorporated with a reconstructed cuff appearance. Clinically, only intermittent residual pain persists, and it is interesting to note that the GRAST does not interfere with the elevation of the arm, which is almost complete (Fig. 6). This is in line with our biomechanical hypothesis on the behavior of GRAST.

After a rotator cuff tear, inactive muscles tend to atrophy and degenerate into fat, visible on Computerized Tomography (CT) and Magnetic Resonance Imaging (MRI) [13]. It would be interesting to evaluate whether restoring continuity between the posterosuperior cuff and the greater tuberosity reactivates the function of an atrophied muscle. Our only clinical case does not allow us to conclude such a recovery, but we have started a clinical study by including patients operated on using the Braided GRAST technique under arthroscopy, by comparing muscle trophicity with imaging of the supraspinatus before and at a distance from surgery, and by evaluating the contractility of the supraspinatus muscle whose continuity with the greater tuberosity has been restored in dynamic ultrasound.

A second limitation is the need to harvest from another anatomical site. As with any graft harvesting, this adds complexity to the technique and increases the risk of specific complications: infection, donor site morbidity, failure of the harvesting, residual pain. This limitation is important as more and more surgeons are over-specialized in shoulder surgery and are therefore unfamiliar with the removal of harmstring tendons. However, the removal of these tendons is commonly performed for cruciate ligament surgery. For surgeons who are used to this procedure, it takes only a few minutes and the scar is short. The rate of infection and specific complications is exceptionally low [7]. The main complication is damage to the infrapatellar branch of the saphenous nerve, generating hypoesthesia distal to the scar. Although frequent, estimated at between 30 and 50% of patients, this complication is extremely well tolerated [9]. Furthermore, the GRA and ST tendons offer a tissue volume that is much greater than that which the upper limb can provide, which explains their varied use for ligament surgery of the upper limb [9, 18]. Finally, these tendons regrow between 60 and 80% of cases in the months following their removal [19].

## Conclusions

In irreparable posterior cuff tears, the tendon defect can be filled by the braided GRAST, which restores the musculo-tendon continuity, and could have a humeral head lowering action and regain at least partially, the motor function of the supra- and infraspinatus and give patients back external rotation. This simple, fast, safe and inexpensive procedure could be an alternative to the proposed treatments, especially in patients not eligible for reverse arthroplasty or tendon transfer. This anatomical study opens the door to clinical experimentation.

## Data Availability

The datasets generated and analysed during the current study are not publicly available due to respect of anonymity and medical data but are available from the corresponding author on reasonable request.

## Abbreviations

GRA: Gracilis
ST: Semitendinosus
GRAST: Transplant composed of gracilis and semitendinosus
SS: Supraspinatus
IS: Infraspinatus
APHP: Assistance Publique des Hôpitaux de Paris
CNIL: Commission Nationale de l’Informatique et des Libertés
CT: Computerized Tomography
MRI: Magnetic Resonance Imaging
